# Feasibility of a smartphone app to enhance physical activity in progressive MS: a pilot randomized controlled pilot trial over three months

**DOI:** 10.7717/peerj.9303

**Published:** 2020-06-23

**Authors:** Navina N. Nasseri, Eghbal Ghezelbash, Yuyang Zhai, Stefan Patra, Karin Riemann-Lorenz, Christoph Heesen, Anne C. Rahn, Jan-Patrick Stellmann

**Affiliations:** 1 Institute of Neuroimmunology and Multiple Sclerosis (INIMS), University Medical Center Hamburg-Eppendorf, Hamburg, Germany; 2 Academy for Training and Career, University Medical Centre Hamburg-Eppendorf, Hamburg, Germany; 3 Universitäres Kompetenzzentrum für Sport-und Bewegungsmedizin (Athleticum) und Institut und Poliklinik für Medizinische Psychologie, University Medical Center Hamburg-Eppendorf, Hamburg, Germany; 4 Department of Neurology, University Medical Center Hamburg-Eppendorf, Hamburg, Germany; 5 Hopital de la Timone, CEMEREM, APHM, Marseille, France; 6 CNRS, CRMBM, UMR 7339, Aix Marseille University, Marseille, France

**Keywords:** Multiple sclerosis, Physical activity, Evidence based patient information, Smartphone, RCT

## Abstract

**Background:**

People with chronic progressive multiple sclerosis (CPMS) have limited options in medical treatment. Enhancing physical activity (PA) might promote neuroregeneration in multiple sclerosis (MS) and positively influence disability, thus providing an alternative to medical treatment. Previous studies indicate that evidence-based patient information (EBPI) is essential for inducing behavioral change, e.g. enhancing PA.

**Objective:**

To investigate feasibility of a smartphone app providing EBPI about the benefit of PA and a simple activity feedback to enhance PA in people with CPMS in a pilot randomized controlled trial over 3 months.

**Methods:**

Thirty-eight people with CPMS (mean age 51 years, median Expanded Disability Status Scale 4.0) were 1:1 randomized into either a control group (*n* = 20) or an intervention group (*n* = 18). The intervention group received access to a multimedia EBPI app including activity feedback, texts, figures and videos. In the control group, participants received a leaflet with unspecific information about exercising in general. The EPBI itself was designed based on a systematic review. At baseline and after 3 months, all participants underwent clinical performance tests, filled in questionnaires and received an activity monitor (Actigraph^®^) for 7 days. The primary endpoint was the rate of responders defined as participants with a 20% increase of physical acitivity (time of moderate or vigiorous PA—MVPA) or 20% increase of the number of steps, both assessed with the activity monitor. As secondary endpoints, we compared accelerometry, performance and questionnaires adjusted for baseline measurments between the groups (ANCOVA). Moreover, we used questionnaires to compare knowledge about exercise (activity requiring physical effort, carried out to improve or improve health and fitness) in MS, usability of the app in general and motivation towards a more active lifestyle after 3 months in both groups.

**Results:**

The groups showed significant differences in disease duration and PA according to the Godin–Leisure Time Exercise Questionnaire at baseline. After 3 months, we detected no difference in the rate of responders, which was an overall 22%. However, MVPA significantly increased in both groups (*p* < 0.001) and the intervention group tended to have a higher motivation towards a more active lifestyle (Cohens *D* = 0.7, *p* = 0.09) as measured by the questionnaire. Reponses also showed, that participants appreciated the app but claimed a lack of interactivity as a short-coming.

**Conclusion:**

Just providing information in a multimedia smartphone app did not enhance physical activitiy more than a simple leaflet in this small pilot trial in CPMS. However, the group of app users tended to have a higher motivation towards a more active lifestyle. Overall, the concept of a smartphone app to support an active lifestyle in MS is highly appreciated by participants.

## Introduction

Multiple sclerosis (MS) is one of the most common disabling neurological diseases of adults. The accumulation of disability is driven by inflammation and neurodegeneration (Aarli et al., 2014; Dendrou, Fugger & Friese, 2015). While there are several treatment options for the relapsing-remitting disease course and its associated inflammatory activity, there is no medication targeting neurodegeneration. As neurodegeneration, opposed to inflammatory activity, makes up the main pathomechanism and correlate for symptoms of persons with progressive or long-standing MS and yet has no direct treatment available, the clinical findings resemble a chronic and less variant disease progression (Ontaneda et al., 2017). A possible treatment approach for progressive or long-standing MS might be lifestyle interventions, particularly those enhancing physical activity (PA) (Motl, 2014). While animal research has sufficiently proven the neuroprotective potential of exercise (Tari et al., 2019), clinical research in humans also indicates the promotion of neuroregeneration and plasticity through exercise (Hötting & Röder, 2013; Reynolds et al., 2018). Prove for these indications is yet to be found amongst ongoing research. PA, defined as any skeletal muscle body movement that results in energy expenditure, extends the concept of exercising to a generally more active life style, including also occupational work, transportation and housework (Motl, 2014). Conceptually, a more active life style depends on all kinds of self-chosen activities and also planning and integration of activities in daily life. Especially short but regular activity bouts rather than selected long high intensity exercises define the difference between the broader reference of the phrase active life style in comparison to the word activity. In this definition walking, as a means of transportation, represents an essential part of managing an anticipated decrease in mobility and an active lifestyle as it is depicted in this study (Motl et al., 2011). It is well known that even in less disabled relapsing remitting MS, only 20% of individuals meet the WHO criteria for an active lifestyle (compared to 40% in the general population) and show a rapid further decrease (Motl, 2014). Specific information about activity levels in progressive or long-standing MS are lacking but can be etimated to be even lower. However, reduced mobility is a key symptom of long-standing or progressive MS (Ebers et al., 2008; Orbach et al., 2012; Kinnett-Hopkins et al., 2017). Moreover, very few studies have adressed exercising effects in progressive or long-standing MS in particular. The few studies found show that enhanced PA through regular exercising over a time frame from a few weeks to 6 months improves cardiorespiratory fitness, muscular strength, endurance, walking ability, cognition, fatigue and health-related quality of life in MS (Romberg, Virtanen & Ruutiainen, 2005; Hebert et al., 2011; McDonnell, Smith & MacKintosh, 2011; Kuspinar, Rodriguez & Mayo, 2012; Paltamaa et al., 2012; Pilutti et al., 2013; Latimer-Cheung et al., 2013; Medina-Perez et al., 2014; Plow et al., 2014; Dalgas, Stenager & Sloth, 2014). As short-term clinical studies researching both exercise and PA in general have already shown feasibility of keeping patients with MS adherent to a treatment regimen, substantial neuroprotecive effects might only be achievable through long-term adherence and behavioral change (Motl, 2014; Casey, Coote & Byrne, 2019).

Evidence-based patient information (EBPI) is increasingly recognized as a useful approach to enable shared decision-making and fulfilling patients´ requests for autonomy and self-management (Hofmann et al., 2013). In parallel with that, internet-based, cognitive-behavioural interventions (Fischer et al., 2015; Pöttgen et al., 2018) have also been proven as effective in MS (Motl et al., 2011; Moss-Morris et al., 2012; Casey, Coote & Byrne, 2019). However, several aspects about the design of complex interventions such as the theoretical framework, are still a matter of discussion (Casey, Coote & Byrne, 2019). In light of these findings, a smartphone-based study can therefore be viewed as the next logical step as the smartphone appears to be the most commonly used device to access to the internet nowadays (Eurostat Press Office, 2016). The flexible usability of a smartphone and the ability to continuously update content are particularly advantageous for users when compared to traditional information-delivery strategies such as brochures or conventional pamphlets. This approach furthermore especially matches the change in information strategies within the last decade towards young adults, who make up the greatest group of people with MS (rapid increase between 20 and 35 years resulting in a peak prevalence at 45 years) (Dilokthornsakul et al., 2016). In addition, the demand for explanatory multimedia content such as videos and animations can be satisfied (Swallow et al., 2014). Another benefit is the resulting low barrier to accessibility for people with MS who have limited mobility or live far away from MS centers or neurological practices.

In terms of content, previous EBPI programs for MS focused in particular on therapeutic decisions, risk management of therapies and early phases of MS (Kasper, Heesen & Mühlhauser, 2009), while information programs for people with long-standing or chronic progressive multiple sclerosis (CPMS) are rare (Heesen et al., 2009, 2010, 2011; Hofmann et al., 2013). The underlying application focuses specifically on information on chronic progressive MS while embedding this in information on MS in general. Another aspect embodied in the application is accelerometry, which offers the possibility to enable the quantification and feed-back of real-life mobility (Stellmann et al., 2015). Through its ubiquitous deployment in smartphones, accelerometry could serve as a feedback mechanism of an app-based intervention program to capture PA. It furthermore ensures objective measures of participants’ activity to validate possible effects of the intervention. Monitoring and enhancing mobility links its well-known loss in pwMS with an essential part of PA that is also commonly adressed in behavioral interventions (Motl et al., 2011; Kinnett-Hopkins et al., 2017). Thus, combining EBPI via an internet-based intervention, an easy accessible feed-back mechanism with contemporary information services that is, movies delivered on smartphones might be a feasible approach to enhance PA in progressive or long-standing MS. In the present pilot-randomized controlled trial (RCT), we aimed to investigate the feasibility and ability of a contemporary internet-based EBPI/feedback smartphone application to effectuate PA in people with chronic progressive MS over 3 months.

## Materials and Methods

### Study design

**Figure 1:**
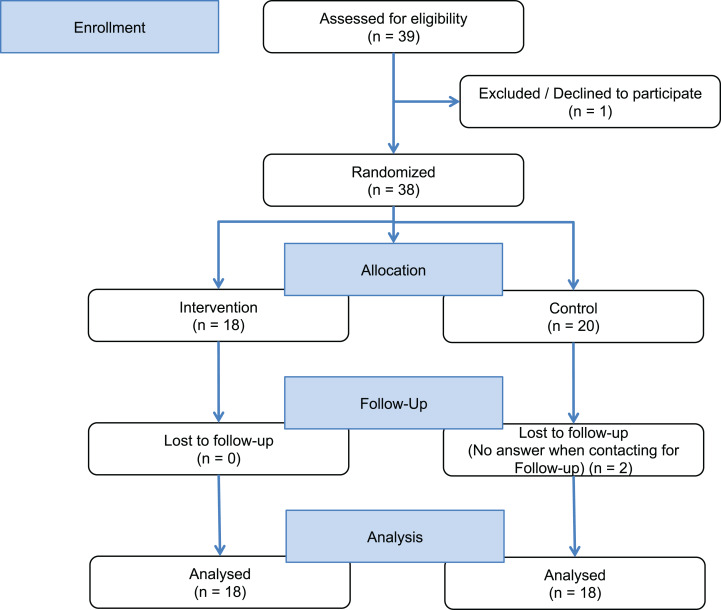
Study flow chart.

We conducted a rater-blinded RCT (NCT03114293) with people with chronic progressive MS 1:1 randomly assigned to the intervention group or a control group. The intervention group received access to a customized mobile app including the EBPI and basic feedback on PA. The control group received a very simple two page leaflet with general information about the health effects of exercising without any EBPI content (see Supplemental Material). Allocation to the intervention or control groups was exercised by handing participants an envelope at baseline (40 opaque envelopes had been prepared, 20 with an access code to the app and 20 with a faux code). Participants of both groups had to meet diagnostic criteria for clinically definite MS with a primary or secondary progressive disease course (Montalban, 2012), mild to moderate disability defined by an Expanded Disability Status Scale score (EDSS) (Kurtzke, 1983) below 6.5 and an age of 18–60 years. Participants were excluded if they had any serious illness other than MS, serious cognitive deficits (known Symbol Digit Modality Test (SDMT) <−2.5 SD or participants who failed to understand the aim or timeline of the study) or serious restriction of upper limb motor skills, which impair the use of a smartphone. All participants received identical smartphones (Samsung-S4 Mini, same production series) with built-in accelerometer and the app, which was only activated in the intervention group. Participants were instructed not to share their accessibility of the application during visits or unpredecented contact with the study conductors (Partipiciants flow chart—Fig. 1).

### Measures and endpoints

At baseline and after 3 months, partipicants underwent a clinical assessment including expanded disability status scale (EDSS) (Kurtzke, 1983), 2 and 6-Min walking tests (2MWT and 6MWT) (Gijbels, Eijnde & Feys, 2011), a Timed Tandem Walk (Stellmann et al., 2014), Five Times Sit to Stand Test (Møller et al., 2012), and the Multiple Sclerosis functional composite (MSFC) (Ontaneda et al., 2012) with Timed 25 Foot walk (T25FW), Nine Hole Peg Test (NHPT) and SDMT. Their weight and waist width were also obtained. Patient reported outcome measures (PROMS) adressed mobility with the motor scale of the “Hamburg Quality of Life Questionnaire Multiple Sclerosis” (HAQUAMS) (Gold et al., 2001) and the MS Walking Scale (MSWS) (Motl et al., 2014). In addition, activities of daily life (Frenchay activity index, FAI) (Schuling et al., 1993) and free time activities (Godin–Leisure Time Exercise Questionnaire, GLTEQ) (Godin & Shephard, 1985) were assessed (all questionnaires as pen and paper version filled out by the participants). Participants received an Actigraph^®^ accelerometer (actigraphcorp.com) to measure PA over 7 days, and we extracted mean steps per minute, daily average metabolic equivalent of task (MET) and the proportion of moderate to vigorous PA (MVPA) during the measurement, listed in percentage. At the end of the study, all participants received a questionnaire tailored to check for group differences concerning the ability to interpret medical information based on the Medical Data Interpretation Test (Schwartz, Woloshin & Welch, 2005), to test their knowledge about exercise in MS and to estimate their motiviation towards a more active lifestyle (lifestyle with higher-than-neccessary amounts of PA). In addition, the participants rated comprehensibility, usability, and other app contents such as pictures and videos on graphical continuous rating scales (1 signifying no comprehensibility respectively usability and 5 signifying the highest possible comprehensibility respectively usability). For this purpose questions were directly addressing the participants’ satisfaction regarding their individual level of overall comprehension, perceived usefulness of the app and its components.

The study was conducted at the MS Day clinic of the university medical center Hamburg—Eppendorf. Recruitment took place between April and December 2016. All assessments were performed under the same conditions and by the same rater (medical students NNN and YZ, trained and supervised by an experienced neurologist JPS) to reduce the variability, especially the known inter-rater variability for the EDSS. Appointments were scheduled after hours to avoid interference with ongoing business and to facilitate participation for full-time employes. Pseudonymized data was stored in an electronic case report form. All participants gave written informed consent prior to study entry and the protocol was approved by the local ethics committee (PVN 5001; Ärztekammer Hamburg, Hamburg, Germany).

The primary endpoint was defined as a higher rate of responders in the intervention group defined by a 20% increase of mean steps per minute or a 20% increase of PA (METs). A sample size of *n* = 36 yielded a power of 80% to detect an effect size of 0.47. With a lack of clinically meaningful cut-offs for our outcomes of interest, we used the best available cut-off from the T25FW. It can be considered the most widely used walking test in MS and it is consensus to accept 20% as a significant change for this test (Motl et al., 2017). Even though we followed a feasibility approach in this study, we included a clinical endpoint as putative strongest read out for feasbility. However, real-life accelerometry is not yet accepted as an endpoint for clinical trials in MS and we included also commonly used clinical outcomes and PROMS. All secondary endpoints were also screened as putative outcomes for a later confirmatory trial and if they are feasible to determine confirmatory sample sizes. These secondary endpoints included improvement of other accelerometer activity measures, as well as the measures introduced above (clinical assessment including MSFC). To evaluate feasibility of the smartphone more specifically, we included the questionnaire about usability, comprehensibility and content rating as additional secondary endpoints. As exploratory outcomes, the effects on the participants’ motivation to adhere to a more active lifestyle in comparison to baseline and other subjective developments were calculated.

### EBPI and app development

The production of the EBPI and of the app followed a predefined development plan: (1) Systematic literature search. (2) Preparation of EBPI. (3) Constructing the patient information app (PIA).

#### Systematic literature search

A systematic literature search in PubMed was conducted using the following keywords: “exercise” OR “PA” OR “rehabilitation” AND “progressive MS” OR “CPMS”. Eligibility criteria: The research was limited to English-language studies and meta-analyses (published between 2000 and 2014) that evaluated the effects of exercising on the following domains: Muscle strength, fitness, mobility, balance, cognition, depression, fatigue, safety and health-related quality of life. Studies without a pure exercise intervention, for example, robotic supported gait programs or complex interventions were excluded. Because most RCTs concerning the above mentioned criteria were conducted on people with relapsing-remitting MS, studies with accordingly classified participants were also regarded for general conclusions on the subject. Further, eligibility criteria for inclusion of RCTs in the review were used as follows:

- Participants with relapsing or chronic-progressive MS, *n* > 35.
- Treatment (aerobic or resistance training) vs. control (no treatment).
- Intervention period >8 weeks.
- Non-randomized and non-controlled pre-experimental studies, studies with a single session design, abstracts and review articles were excluded.

**Figure 2:**
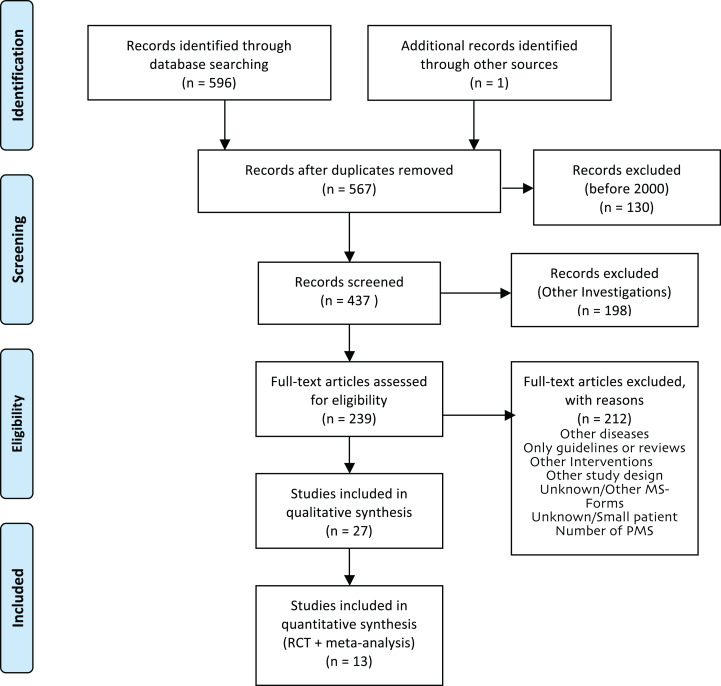
PRISMA flow diagram of literature review process for the EBPI.

For the production of the EBPI, we aimed to identify the most recent meta-analysis for each domain, if available, and added studies published afterwards. Two researchers (EG, JPS) screened 437 titles and abstracts to identify RCTs and meta-analyses. Out of the resulting 239 full-text articles, six meta-analyses that fulfilled the inclusion criteria were identified and served as the basis for the EBPI. The reference lists of these 6 publications as well as the authors’ personal databases were checked for further relevant publications that were not captured by the initial search, because the effect of PA on some symptoms such as balance or quality of life in meta-analyses was only marginally investigated. The search for current relevant RCTs not yet included in the meta-analyses or not covering the above mentioned domains identified seven further RCTs. Finally, a total of 13 studies (meta-analyses and RCTs) were selected for inclusion in the EBPI (Fig. 2).

#### Preparation of EBPI in German language

The EBPI was written (EG) to describe the evidence concerning the effect of exercising along the following domains: Muscle strength, fitness, mobility, balance, cognition, depression, fatigue, safety and health-related quality of life. Further topics lacking RCT data included effects on MRI and physiology and were reported and clearly labeled as expert opinion. Table 1 summarizes the key information for each domain of the EBPI. Figures, texts and the structure of the EBPI followed EBPI developement pathways established in recent years at the institute of neuroimmunology and MS. All content was edited by JPS, CH and AR to guarantee readability (Heesen et al., 2004, 2009; Kasper et al., 2006; Kasper, Heesen & Mühlhauser, 2009; Köpke et al., 2009; Hofmann et al., 2013; Brand et al., 2014).

**Table 1:** Summary of key information about exercising effects in the EBPI.

Strength and fitness	• A total of 54 studies with 900 participants • Strength improves • Endurance improves • Normal training for patients with MS possible	Latimer-Cheung et al. (2013)
Mobility	• A total of 25 Studies with 80 participants • Improvement through sport • Longer walking • Faster walking	Latimer-Cheung et al. (2013)
Cognition	• Only two studies with 77 participants • Improvement of reaction speed • No proven effect on learning or memory	Briken et al. (2014)
Balance	• A total of 6 studies with 230 participants • Individual balance training may be more effective than strength or endurance training alone.	Paltamaa et al. (2012)
Depression	• A total of 12 studies with 476 participants • Exercising improves mood • Exercising may protect against depression	Dalgas, Stenager & Sloth (2014)
Fatigue	• A total of 45 studies with 2250 participants • Exercising reduces fatigue • Clearly no deterioration	Heine et al. (2015)
Quality of life	• A total of 39 studies with over 2900 participants • Improvement of quality of life • Superior to sole symptomatic therapies	Kuspinar, Rodriguez & Mayo (2012)
Yoga	• A total of 7 studies with over 670 participants • Yoga improves depression and fatigue significantly • Yoga tends to improve mobility and quality of life	Cramer et al. (2014)
Safety	• No negative impact on relapse rate • No increased risk of injuries during endurance sports • Questionable minimal increased risk for injuries with pure strength training • Uhthoff	Pilutti et al. (2014)

#### Constructing the mobile app

A private company was engaged which set up a content management system (CMS) to develop the app. The CMS was then equipped with a total of 50 diagrams and about 25 short film clips. Videos contained short interviews with different medical staff such as neurologists, physiotherapists and MS-patients sharing their experience with exercise. Overall, the CMS was analogously structured as a common wiki with embedded figures and videos with options for adapting font size and luminositiy. The final EBPI contained 385 pages, it provided comprehensive information on various forms of training, their correct implementation, the risk of adverse events and side effects. Due to the similarity to common internet-based information systems, individual training on how to use the EBPI was not implemented.

For activity feedback, we used the not yet validated approach of calculating the smartphone accelerometry data (vector magnitude, VM, that is, the square root of accelerations over all three axes) (Yang & Hsu, 2010). The app presented a graphically processed statistic with monthly, weekly and daily mean values of VM as a barplot where the *x*-axis represented the time and the hight of the bars represented the average value for the bouts. The PIA-app of our study was created using the Android programing language and matching applets in the most suitable version. Two important aspects were taken into account: Content management usability for timely content updates and protection of user data.

### Statistics

Statistical analyses were done using “Statistics in R”. Besides descriptive statistics, we compared groups at baseline with Chi Square or Student’s *t* test (Wilcox test for the ordinally scaled EDSS). We computed changes from baseline to follow-up for each outcome. For the primary endpoint an increase of ≥20% for steps or PA (METs) defined a responder and groups were compared via Chi Square test. For all other outcome measurements, we used ANCOVA corrected for baseline values to evaluate group differences. Besides per protocol results, we performed intention-to-treat analyses. An alpha level below 0.05 was considered statistically significant.

## Results

### Demographics

The intervention group had a shorter disease duration (mean: 13.1 years, controls: 20.1 years, *p* = 0.04) and showed higher activity levels at baseline according to GLTEQ (mean: 27.3 points, controls: 13.1 points, *p* = 0.03) than controls. Other measures taken at baseline showed no significant difference between study groups (Tables 2 and 3).

**Table 2:** Demographics. Data as mean (sd) if not otherwise indicated.

	Intervention	Control	*p*-Value
	*N* = 18	*N* = 20	
Sex (female/male) *n*	9/10	11/9	0.87
Age (years)	49.6 (8.5)	52.5 (7.3)	0.26
Weight (kg)	78.7 (16.3)	72.1 (18.2)	0.25
Waist (cm)	97.1 (13.5)	94.0 (17.5)	0.55
Disease duration since first symptoms (years)	13.1 (5.6)	20.1 (13.0)	0.04*
EDSS median (range)	3.5 (2.5–6.0)	3.5 (3.0–6.0)	0.30

**Note:**

* *p* < 0.05.

**Table 3:** Primary and secondary endpoints at 12 weeks. Summary for primary and secondary outcomes at baseline and follow-up: Absolute values, absolute difference between visits, difference of means between groups at follow-up and partial eta squared as effect size estimate from ANCOVA. Data as mean (sd) if not otherwise indicated.

	Intervention	Control	*p*-value
	*N* = 18	*N* = 20	
Primary endpoint			
Responder *n* (%)	4 (22%)	7 (37%)	0.47

Secondary endpoints change from baseline mean (sd)	Baseline	Follow-up	Change from baseline			Baseline	Follow-up		Change from baseline		Baseline differences *p*-value	Difference of means (control-intervention) at follow up [95% CI]	Follow-up ANCOVA eta (*p*-value)
EDSS median (range)	3.5 (2.5−6.0)	3.5 (2.5−6.0)	+0.2 (0.4)			3.5 (3.0−6.0)	4.5 (2.5−6.5)		+0.3 (1.1)		0.30	0.3 [−0.29 to 0.89]	0.04 (0.28)
Clinical tests													
T25FW (s)	6.5 (3.6)	5.3 (1.8)	−0.6 (1.5)			7.3 (3.9)	6.3 (2.1)		0.0 (1.6)		0.51	0.79 [−0.15 to 1.73]	0.09 (0.10)
TTW (s)	13.5 (5.8)	12.5 (5.2)	−0.1 (3.1)			13.8 (3.5)	13.5 (4.0)		+0.1 (4.7)		0.87	0.41 [−2.47 to 3.29]	<0.01 (0.77)
2MWT (m)	154 (45)	159 (38)	−14 (20)			139 (34)	143 (31)		+14 (20)		0.27	−3.59 [−19.77 to 12.58]	0.01 (0.64)
6MWT (m)	447 (134)	465 (109)	−8 (55)			393 (120)	430 (92)		+14 (43)		0.22	13.79 [−24.48 to 52.06]	0.02 (0.45)
9HPT dominant (s)	22.9 (4.7)	22.1 (4.0)	−0.6 (2.5)			25.1 (5.9)	25.2 (5.7)		−0.4 (3.5)		0.22	0.77 [−1.12 to 2.67]	0.02 (0.40)
9HPT non dominant (s)	23.1 (3.3)	22.0 (2.7)	−0.9 (1.7)			25.3 (5.0)	25.5 (6.9)		+0.1 (4.6)		0.12	0.98 [−1.44 to 3.39]	0.02 (0.40)
FTSTST (s)	13.6 (6.4)	13.0 (6.2)	−0.6 (1.9)			14.2 (4.6)	12.3 (4.0)		−0.8 (2.9)		0.76	−0.23 [−2.01 to 1.56]	<0.01 (0.80)
SDMT	51.4 (10.9)	48.2 (10.3)	−2.7 (8.8)			44.3 (13.0)	45.0 (9.8)		−0.3 (8.7)		0.07	0.16 [−5.17 to 5.48]	<0.01 (0.95)
7-day accelerometry													
Steps per minute	7.3 (2.8)	6.7 (2.7)	−3.6 (3.6)			5.8 (2.3)	7.0 (2.4)		+0.9 (2.3)		0.12	0.53 [−1.29 to 2.35]	0.01 (0.54)
Mets per day	1.4 (0.2)	1.4 (0.2)	−0.04 (0.26)			1.3 (0.2)	1.4 (0.2)		+0.05 (0.13)		0.17	0.03 [−0.09 to 0.16]	0.01 (0.56)
% MVPA	11.0 (5.2)	17.6 (7.1)	+6.5 (7.3)			9.3 (5.0)	19.9 (7.3)		+7.2 (9.0)		0.34	2.9 [−1.92 to 7.75]	0.05 (0.22)
PROMS													
GLTEQ	27.3 (16.9)	25.4 (20.1)	−2.3 (11.0)			13.1 (18.4)	19.7 (13.7)		+4.4 (15.3)		0.03*	3.04 [−6.81 to 12.90]	0.02 (0.51)
MSWS	30.6 (10.9)	31.9 (12.4)	+1.8 (6.6)			36.6 (13.3)	38.2 (11.0)		−0.3 (8.3)		0.16	0.09 [−0.42 to 0.60]	<0.01 (0.82)
FAI	39.1 (9.0)	(36.1) 12.4	−2.9 (6.9)			36.3 (6.8)	34.5 (7.2)		−1.1 (6.3)		0.33	−0.58 [−6.17 to 5.00]	0.02 (0.50)
HAQUAMS motor scale	2.4 (0.7)	2.5 (1.0)	+0.2 (0.7)			2.7 (0.7)	2.9 (0.7)		+0.1 (0.7)		0.15	1.73 [−3.56 to 7.01]	0.01 (0.71)

**Note:**

* *p* < 0.05.

### Clinical endpoints and PROMS

**Figure 3:**
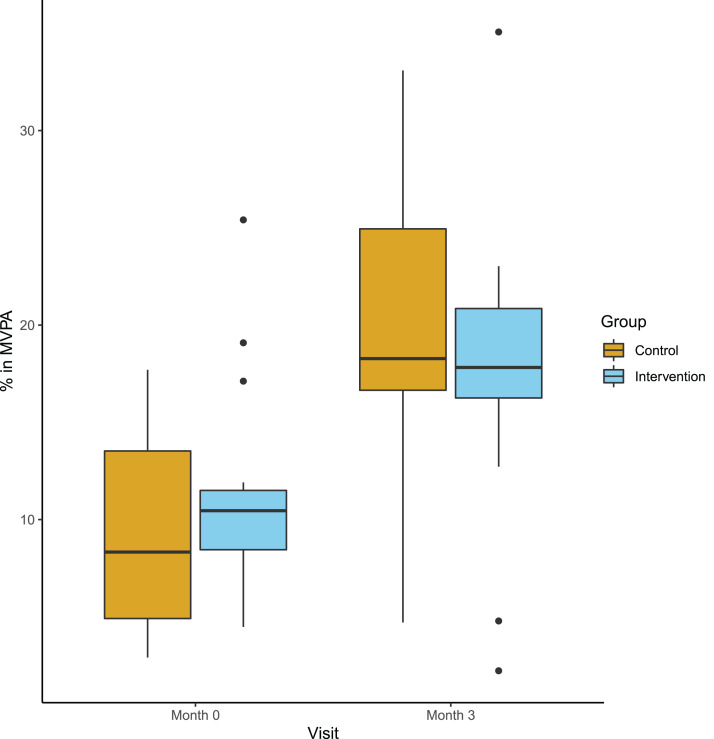
Percentage of moderate or vigiorous physical activity at baseline and follow-up. Boxplot (median and Quartals) with whiskers representing the 95% CI of the median

The primary endpoint, defined as the responder rate (20% increase in steps or MET) did not differ between the groups: In the intervention group four out of 18 participants, compared to seven out of 18 in the control group (*p* = 0.47) could be classified as responders. Secondary clinical endpoints adressed group differences at follow-up corrected for baseline values. Table 3 summarizes mean values at both time points, mean absolute changes and mean differences at follow-up and the corresponding *p*-values. We observed an increase of MVPA in both groups from baseline to follow-up, but no difference between the intervention and the control group (Fig. 3). Other actigraph metrics and all clinical performance tests remained stable in both groups and did not show any group differences. Similariliy, we observed no group differences in PROMS adressing perceived mobility, PA and activities of daily living. Here, we report the per protocol results. Intention-to-treat analyses did not differ.

### Knowledge, motivation and app rating

General questions testing the participants’ overall ability to understand statistical data and deriving correct conclusions were answered by both groups with a similar success (*p* = 0.94). The knowledge about safety and efficacy of exercising in MS was similar for both groups after three months, but rather poor. The intervention group had 5.9 out of nine possible points and the control group scored an average of 5.4 points (*p* = 0.68). When asked if participants in general felt motivated to start and consecutively maintain a more active lifestyle in the close future, the intervention group showed a tendency towards higher levels of motivation (mean: 4.8) than controls (mean: 3.8, Cohen’s *d* = 0.7, *p* = 0.09). The intention to change one’s behavior towards a more active lifestyle was equally high in both groups (4.9 vs. 4.8 on a six point Likert scale, *p* = 0.94).

**Figure 4:**
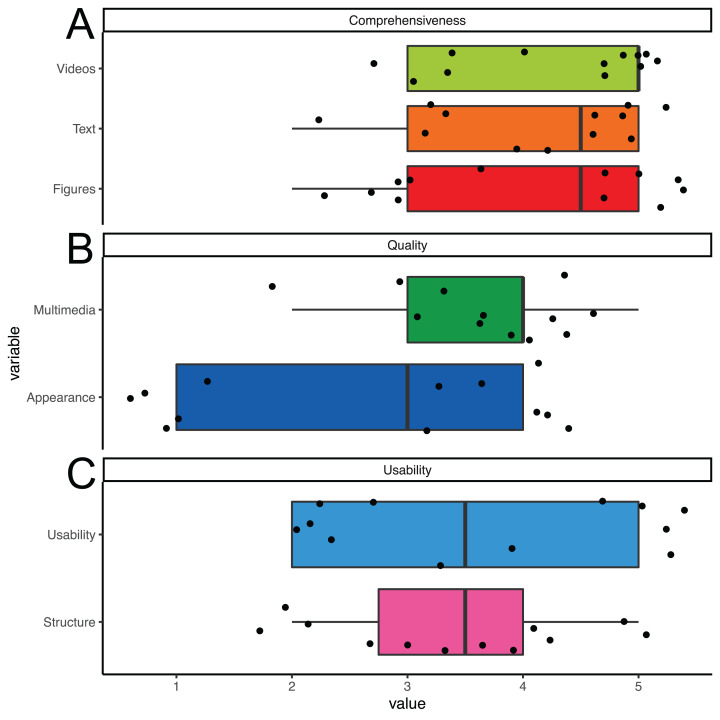
App rating. Boxplot (median and Quartals) with whiskers representing the 95% CI of the median. Single values jittered. Users rated (A) comprehensiveness, (B) quality and (C) usability with higher scores representing better ratings.

The overall usability of the PIA app was rated in seven questions about understandability and general usefulness on a continuous scale ranging from 1 = “not at all” to 5 = “absolutly” with a mean value of 3.7 points as helpful indeed (Fig. 4). The perceived support of the application in helping towards a more active lifestyle was estimated at four out of six points. Reported technical issues when using the app were such as a short battery life, occasional auto shut off of the app or phone and the feedback monitor failing to refresh according to a participants actual acitivity level. When given the opportunity to express their requirements for a good mobility feedback monitor with EBPI, participants requested more hyperlinks to relevant websites, more pictures and videos and more texts. Moreover, several participants demanded a more interactive format. In a feedback box, participants mentioned that the PIA app helped them in making lifestyle changes and the app supported their way of living.

## Discussion

This study investigated the feasabilty of a smartphone based multimedia approach to enhance PA in people with chronic progressive MS. Overall, participants appreciated the approach and found the app both easy to use and helpful towards a more active lifestyle. However, after three months the PA assessed with accelerometry did not differ between the app users and the control group in this small study. However, app users tended to be more motivated. Interestingly, we observed an increase in MVPA in participants from both groups.

### High acceptance of smartphone app for pwMS

In MS, the need for up-to-date information is high and an increasing number of people with MS already use electronic communication methods to access health related information, connect with fellow patients and their health care providers (Haase et al., 2012). Here, we designed a contemporary app-based EPBI including multimedia content such as expert videos and testimonials. The acceptance of our app is in line with previous research, indicating a high approval of internet-based solutions in MS—especially if the users report previous experience with electronic resources (Apolinário-Hagen et al., 2018). However, the intervention failed to indicate a tendency of physical activitiy enhancement in people with progressive MS in comparison to a simple two-page leaflet. Also, the knowledge about exercising effects in MS was identical in both groups.

### Interaction as a key feature of app based interventions

One of the most commonly reported short-comings concerning our app was the lack of interactive features. The very simple and not validated activity feedback provided in the app cannot be rated as a tailored feed-back mechanism. This view of the participants is supported by several studies, indicating that efficacy of inducing behavioral change is greatest in multimodal approaches as opposed to standalone app interventions. For higher success rates they would have to include cognitive behavioral therapy or change strategies which were missing in our small study (Schoeppe et al., 2016; Casey, Coote & Byrne, 2019). Previous research adressing PA in MS showed an increased impact of behavioral change interventions if combined with an internet based approach (Motl et al., 2011). Others indicated that empowerment and knowledge might be independent of interactive features in eHealth applications (Camerini & Schulz, 2012). However, future editions of our app should include a guided behavioral intervention (Casey, Coote & Byrne, 2019).

### Inclusion of digital natives beneficial for future studies

Due to our focus on progressive MS thus targeting older individuals (due to the peak prevalence of PPMS/SPMS at the age of 40 years), most participants originate from non digitally native generations and thus might have less experience and/ or interest in using their smartphone regularly than younger MS groups. It is known that previous experience with electronic communication increases the acceptance for eHealth and internet-based approaches (Apolinário-Hagen et al., 2018). Thus, future studies might expect larger effect sizes, as they will naturally comprise higher numbers of “digital natives” and therefore greater general susceptibility for mobile applications.

### Increased PA is independent from allocated cohort

Interestingly, the amount of moderate to vigorous PA increased significantly in both groups based on the accelerometer, while this change was not reflected in the PROMS. This discrepancy might be explained by the previously shown tendency towards overestimation of PA in self-reporting settings compared to objective measures (Duncan et al., 2001; Troiano et al., 2008). As the reliability of the accelerometer has been researched and proven (Aadland & Ylvisåker, 2015) we interpret the accerlerometric findings as more reliable than PROMS findings in the context of our study. An underlying overestimation might also have been the cause for above mentioned large differences at baseline. This might also explain the consecutive decrease of GLT reported activity in the intervention group. Partipicants of this group might have been more realistic at follow-up. Moreover, simple changes in real-life behavior such as using stairs instead of elevators are not detected by GLT, but are considered to be important for an active life style. In conclusion some PA increases were detected by the accelerometer but could not have been detected by the GLT. Overall our findings indicate that short interventions like those of our study might have an effect towards a more active lifestyle. Even only briefly mentioning positive effects of exercising in MS in the routine counseling of people with MS might have a reasonable effect.

### Limitations

Besides the lack of interactive features and a structured behavioral intervention, other aspects of our study limited the chance to detect group differences. First, by chance the randomization led to significant differences in the composition of the two groups, such as the control group had a longer disease duration and a lower self-reported activity level. Secondly, the small sample size of this pilot study was only capable of detecting large effects. When responding to the question concerning the magnitude of motivation to begin a new PA, stick to their new PA and still being active several months after the study, the intervention group reached a higher cumulative score than the control group representing a moderate to large effect size of Cohen’s *d* = 0.7, but without reaching statistical significance. However, we did not measure the motivation at baseline and we cannot estimate the true effect on motivation. In addition, the short follow-up time might also explain the missing effects on clinical performance tests such as walking speed. Even under supervised exercising, a time frame of three months might be too short to improve clinical performance test in less disabled MS participants (Baquet et al., 2018). Lastly, the EBPI was constructed based on the available literature about exercising in MS. Most of these studies included only relapsing-remitting MS and our EBPI might have missed specific needs and concerns of the older and more disabled progressive MS populations. However, even performance estimates for semi-professionals are often extrapolated from young adults and do not take age and sex differences into account sufficiently (Huebner, Meltzer & Perperoglou, 2019). As long as there are no specific studies available, the communication of the available knowledge seems acceptable.

### Conclusions and recommendations for future studies

Overall, this small pilot study proved the feasibility of our approach and supports the current strategy to establish electronic behavioral interventions in MS. Our study provides some useful findings for future confirmatory trial (Kaur et al., 2017). Even in this already moderately impaired cohort, we observed a high acceptance of smartphones as a platform for delivering such an intervention and for monitoring the disease. Moreover, our study indicates the special usefulness of smartphones as they might integrate direct feedback on PA and allow interaction with the user during the whole day. For future studies larger cohorts along with a longer duration (≥6 months) might be beneficial towards reaching a significant increase of the everyday PA in people with MS. Conceptually, this endpoint seems to be the most valuable as clinical performance and disability outcomes lack ecological validity and are probably not predictive for real-life changes. However, based on this study, we cannot define a best endpoint or provide a reliable sample size estimate for a confirmatory trial. At last, the fast recruitment in a single center indicates that recruitment for a larger trial will be feasable in a multicenter setting within a reasonable time frame. Randomization should be adjusted for baseline PA reducing the risk for baseline disbalances as observed in our cohort after randomization.

## Conclusions

Just providing information in a multimedia smartphone app did not enhance physical activitiy more than a simple leaflet in progressive MS. However, the group of app users tended to have a higher motivation towards leading a more active lifestyle. Overall, the concept of a smartphone app to support an active lifestyle in MS is highly appreciated by participants.

## Supplemental Information

10.7717/peerj.9303/supp-1 Supplemental Information 1 Trial protocol.

10.7717/peerj.9303/supp-2 Supplemental Information 2 Baseline and follow-up data.

10.7717/peerj.9303/supp-3 Supplemental Information 3 CONSORT checklist.

10.7717/peerj.9303/supp-4 Supplemental Information 4 Information leaflet for the control group. Short information leaflet that served as the control condition in this pilot RCT.

## Additional Information and Declarations

### Clinical Trial Registration

The following information was supplied regarding Clinical Trial registration: NCT03114293

### Data Availability

The following information was supplied regarding data availability: The visit data are available in the Supplemental Files.
