# Supplementary material for: Feasibility of a smartphone app to enhance physical activity in progressive MS: a pilot randomized controlled pilot trial over three months
Source: PeerJ. 2020 Jun 23;8:e9303. doi: 10.7717/peerj.9303 (PMC7319035; doi:10.7717/peerj.9303)
Supplement: Supplemental Information 1 [file peerj-08-9303-s001.pdf]

PIA: Evidence Based **P**atient **I**nformation and Smartphone Accelerometry to Enhance Physical **A**ctivity in MS

and

xPIA: Cross-sectional Study to Validate Smartphone Accelerometry as Monitoring Tool for real-life Physical Activity and Mobility in MS

Study Protocol

**Title**	Evidence Based Patient Information and Smartphone Accelerometry to Enhance Physical Activity in MS
**Short title**	PIA / xPIA
**German Titel**	PIA: Randomisierte Studie zur Verbesserung der körperlichen Aktivität durch eine evidenzbasierte Patienten-Informations-App und Smartphoneakzelerometrie zur Messung der Aktivität für Personen mit chronischer MS<br><br>xPIA: Einwöchige Validierung der Smartphoneakzelerometrie: Körperliche Aktivität und Mobilität im Alltag
**Protocol Version**	2.0
**Protocol Date**	07.07.2015
**Study type:**	Cross-sectional cohort study (xPIA) and a randomised, waiting-group controlled phase 2a feasibility trial (PIA)

Principal Investigator

Name: Jan-Patrick Stellmann, MD
Address: Institute of Neuroimmunology and MS (INIMS)
University Medical Center Eppendorf
Martinistraße 52, 20246 Hamburg, Germany
Phone 0049 40 7410 54076
Fax: 0049 40 7410 56973
E-mail: j.stellmann@uke.de

Herewith, I confirm that I have read the study protocol carefully and declare my consent with it. I will treat and examine the patients in accordance with the study protocol, the national applicable laws, the international guidelines on good clinical practice (ICH-GCP), and the declaration of Helsinki.

Signature:........................ Date: 7.7.15...................
Dr. Jan-Patrick Stellmann

PRINCIPAL INVESTIGATOR

Other INIMS investigators

Name: Prof. Dr. Christoph Heesen, MD
E-mail: heesen@uke.de

Name: Anne Rahn
E-mail: anne.rahn@uni-hamburg.de

Background and Rationale

Evidence-based patient information (EBPI) is an established part of the treatment concept in Multiple Sclerosis (MS) and is a basis for patients' engagement in disease management [1–4]. In contrast to classic brochures or seminars, web-based information databases can easily be updated and can be used more flexible by patients. A „wiki-system" is already implemented in our multicenter trial DECIMS throughout Germany (Decision Coaching in MS – part of the KKNMS Kompetenznetz MS). [5]. Further on, videos, animations or audio files are simple to integrate. Audio-visual media are requested by patients and care givers [6] and improve knowledge transfer and learning in addition to text based content [7]. Especially videos and animations support the understanding of complex issues, if they are not simply consumed but processed in a learning environment and if they are embedded in an overall knowledge acquisition concept of patients [8]. Up to now, EBPI programs were tailored to support decision-making and improve risk management in early phases of the disease. EBPI for patients with progressive MS are still lacking. [1,2,4,9]

Although it has been proposed that overall progressive MS follows a more severe course than relapsing-remitting MS (RRMS), the disease course might be heterogeneous as in relapsing-remitting patients [10]. Disease modifying therapies have seldom been studied, not approved for primary-progressive MS and have high risk low benefit profile in secondary-progressive MS [11]. Yet symptomatic treatments to improve mobility, bladder function and spasticity are available [12,13] Current treatment concepts are based on increased physical ability as regular exercise training is seen as a putatively neuroprotective or even restorative strategy [14–16]. A randomized-controlled trial from our group could already proof that even patients with relevant disability (progressive MS, EDSS 4-6.5) are able to perform a regular exercise training and do not differ from healthy persons in terms of training progress and sport physiology [17]. Patients showed a significant

improvement concerning mobility as well as in objective neuropsychological tasks. Higher physical activity is associated with better quality of life, less depression and fatigue as well as improved cognitive performance [17]. The necessity for more reliable data from observational trials on the natural course including prognostic factors as well treatment trials in progressive MS was recently addressed by the research agenda of „The International Collaborative on Progressive MS“ [18]. Unfortunately, a reliable and ecologic valid measurement of mobility and physical activity is not yet available, as clinical tests do not reflect real life mobility sufficiently [19]. Pedometers and accelerometers are more and more used to measure real life conditions [20–22], but an international standard has not been developed so far. Furthermore, it has not been studied how far using accelerometers implemented in standard smartphones is feasible. A cross-sectional comparison of standard accelerometry and smartphone accelerometry in a MS cohort of different disability levels and healthy controls is a feasible way for validation of such a new outcome.

Due to their ubiquitary distribution, smartphone accelerometry might serve as an easy way/possibility to use feedback mechanism in an app-based intervention program to increase physical activity. Internet-based cognitive-behavioral interventions have been explored to be effective in the last years [16,23,24]. In addition, smartphone-based mobility assessment and intervention might be a promising approach in other MS types and for real-life mobility assessment in observational and interventional trials. While exercise trials up to six months have shown promising findings in MS, long-term adherence is an unresolved issue. Smartphone tools might offer a substantial step forward. Beside their daily usage, their feedback and reminder functions totally integrated in normal lives, they offer the opportunity of having a game-like set-up. Gamification has been discussed as a promising approach for physical activity adherence interventions. This project will deliver a first step in this direction.

Due to the minimal relapse risk and the short-term stability, patients with progressive MS might be the best suitable cohort for short, explorative phase-2a studies of behavioral interventions. In addition,

accelerometry is a promising outcome and monitoring tool for future phase 2 and 3 studies in progressive MS and might help to develop agents with neuroprotective mechanisms of action.

The development of an EBPI program for progressive MS is an unmet need so far. As the EBPI is based on a smartphone app, it can easily be distributed - a benefit for possibly also substantially disabled patients living in rural areas without any specialized MS-centres nearby.

Objectives

- Development of an evidence-based patient information app for progressive MS
- To compare and explore smartphone accelerometry with standard accelerometry tools in MS and healthy controls
- Compare physical activity in a pilot RCT between brochure information and EBPI-APP
- Evaluation of smartphone accelerometry as a mobility feed-back tool

Hypotheses

- Smartphone accelerometry is comparable to standard accelerometry tools (actigraph)
- Smartphone accelerometry differs between MS and healthy controls as well as between different MS disability levels
- A 3-month smartphone based EBPI and accelerometry feedback improves real-life physical activity compared to an information leaflet group

Explorative cross-sectional Endpoints

- Correlation between smartphone accelerometry and actigraph measures (activity counts and steps)
- Correlation between disability and smartphone accelerometry
- Differences between healthy controls and MS patients in smartphone accelerometry

RCT Endpoints

Primary endpoint:

- Rate of responders as defined by a 20% increase of steps or 20% increase in physical activity as measured with the actigraph[25-27]

Secondary endpoints:

- Physical activity and real-life mobility: 1-week accelerometry (actigraph: total activity, distance per day and number of steps) [18,19]

- Smartphone accelerometry
- Physical capacity: 2 and 6 Minutes Walking Test [25]
- Patient reported outcome measures – PROMS:
  - Quality of Life (HAQUAMS) [26]
  - Mobility (MS Walking Scale MSWS-12) [27]
  - ADL (Frenchay ADL) [28]
  - Godin-Leisure-Time [21]

Explorative Endpoints:

- User time in App
- Content rating
- Multiple Sclerosis Functional Composite - MSFC [29]
- Expanded Disability Status Scale - EDSS [30]

Data management

Data will be collected on paper based source documents, pseudonymized and afterwards stored in an approved electronic database. Mobile accelerometry data is pseudonymized twice and transferred by the app to a secure server using a secured network connection. Data transfer from the app will be approved by the data protection official of Hamburg.

Study design and sample size

In a **cross-sectional cohort**, 100 MS patients and 50 healthy controls will wear a standard accelerometer (actigraph) and a smartphone logging the accelerometry data for one week. Additional assessments include clinical gait tests, neurologic examination and patient reported outcome measures. The sample size will allow performing explorative analyses at different disability levels.

For the **RCT**, n=40 patients will undergo a baseline assessment and be randomized to either the brochure waiting group (control group) or app-EBPI (intervention). All patients will receive a smartphone with an accelerometer and the app. Over 12 weeks, EBPI and the accelerometry feedback will only be visible in the intervention group; afterwards it will be visible for all patients. Follow-up and evaluation of the primary endpoint will be scheduled after three months. An open follow-up over another nine months will be smartphone based without clinical visits. A 20% improvement has been discussed to be clinically meaningful concerning

clinical tests [25]. Similar cut-off values for the number of steps measured by accelerometry can be extracted from the literature. Motl et al. found an increase of 800 steps per day as minimally clinically relevant in MS and described a mean number of steps of approximately 4000 per day in a cohort of progressive MS patients [27]. 36 patients will have 80% power to detect an effect size of 0.47 e.g. detect a higher response rate (20% increase of steps or activity counts) of 0.7 in the intervention compared to 0.2 in the control group at a one-sided significance level of 0.05. Assuming two drop-outs per center 40 patients will be needed.

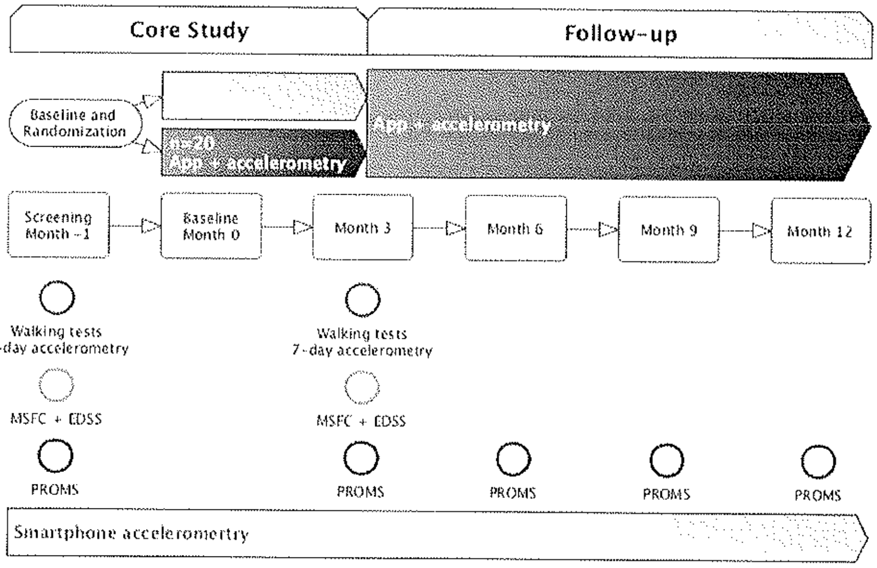

Statsitical analysis plan

Beside descriptive statistics of the cohort and the RCT-population the following analyses will be performed:

Cross-sectional Data: Association between Smartphone accelerometry and actigraph measures will be assessed by regression analyses corrected for gender and age. Differences between healthy controls and MS as well as differences between disability levels will be investigated by t-tests or anova.

RCT: A 20% increase of number of steps or 20% increase of activity counts from baseline to month three will be used to define patients with and without an increased physical activity. Differences between groups are tested with a one-sided (intervention greater than control) Chi-square-test at a significance level of 0.05. Secondary endpoints will compare relative changes from baseline to month three by t-tests.

Analysis of accelerometry data and outcomes will be raterblinded.

1. Heesen C, Kleiter I, Nguyen F, et al. Risk perception in natalizumab-treated multiple sclerosis patients and their neurologists. Mult Scler. 2010 Dec;16(12):1507–1512.

2. Heesen C, Solari A, Giordano A, Kasper J, Köpke S. Decisions on multiple sclerosis immunotherapy: new treatment complexities urge patient engagement. J Neurol Sci. Elsevier B.V.; 2011 Jul 15;306(1-2):192–197.

3. Heesen C, Kasper J, Schäffler N, et al. Übersicht Shared Decision Making in der Neurologie – wieso ? wann ? wie viel ? Neurol Rehabil. 2012;18(4):207–215.

4. Hofmann A, Stellmann J, Kasper J, et al. Long-term treatment risks in multiple sclerosis: risk knowledge and risk perception in a large cohort of mitoxantrone-treated patients. Mult Scler. 2012 Oct 4;

5. Multiple Sklerose: Studie zur Versorgungsforschung | Kompetenznetz MS [Internet]. [cited 2014]. Available from: http://www.kompetenznetz-multiplesklerose.de/de/kohorte-studien-infrastruktur/decims

6. Swallow VM, Hall AG, Carolan I, et al. Designing a web-application to support home-based care of childhood CKD stages 3-5: qualitative study of family and professional preferences. BMC Nephrol. BMC Nephrology; 2014 Jan;15(1):34.

7. Falke T. Audiovisuelle Medien in E-Learning-Szenarien. Formen der Implementierung audiovisueller Medien in E-Learning-Szenarien in der Hochschule–Forschungsstand. In: Apostolopoulos N, Hoffmann H, Mansmann V, Schwill A, editors. E-Learning 2009 Lernen im Digit Zeitalter. Münster, New York, München, Berlin; 2009. p. 223–224.

8. Salomon G. Television is „easy" and print is „though": The differential investment of mental effort in learning as a function of preceptions and attributions. J Educ Psychol. 1984;76:655.

9. Heesen C, Schäffler N, Kasper J, Mühlhauser I, Köpke S. Suspected multiple sclerosis - what to do? Evaluation of a patient information leaflet. Mult Scler. 2009 Oct;15(9):1103–1112.

10. Stellmann J-P, Neuhaus A, Lederer C, Daumer M, Heesen C. Validating predictors of disease progression in a large cohort of primary-progressive multiple sclerosis based on a systematic literature review. PLoS One. 2014 Jan;9(3):e92761.

11. Hartung H-P, Gonsette R, König N, et al. Mitoxantrone in progressive multiple sclerosis: a placebo-controlled, double-blind, randomised, multicentre trial. Lancet. 2002;360(9350):2018–2025.

12. Goodman AD, Brown TR, Edwards KR, et al. A phase 3 trial of extended release oral dalfampridine in multiple sclerosis. Ann Neurol. 2010 Oct;68(4):494–502.

13. Cameron M, Fitzpatrick M. Dalfampridine improves walking speed, walking endurance, and community participation in veterans with multiple sclerosis: a longitudinal cohort study. Mult Scler 2014 May;20(6):733-8.

14. Hartung H. Multiple Sclerosis Impact of Mobility Impairment in Multiple Sclerosis 1 – Healthcare Professionals ' Perspectives. Eur Neurol Rev. 2011;6(July 2010):110–114.

15. Pike J, Jones E, Rajagopalan K, Piercy J, Anderson P. Social and economic burden of walking and mobility problems in multiple sclerosis. BMC Neurol. 2012 Sep 18;12(1):94.

16. Motl RW, Dlugonski D, Wójcicki TR, McAuley E, Mohr DC. Internet intervention for increasing physical activity in persons with multiple sclerosis. Mult Scler. 2011 Jan;17(1):116–128.

17. Briken S, Gold S, Patra S, et al. Effects of exercise on fitness and cognition in progressive MS: a randomized, controlled pilot trial. Mult Scler. 2014 Mar;20(3):382–390.

18. Fox RJ, Thompson A, Baker D, et al. Setting a research agenda for progressive multiple sclerosis: The International Collaborative on Progressive MS. Mult Scler. 2012 Aug 23;

19. Stellmann J-P, Neuhaus A, Stolle C, et al. Physical activity in multiple sclerosis: association of 3D-accelerometry data with subjective and rater-based disease status. Mult Scler. 2011;17(10 suppl):S13.

20. Motl R, Sandroff B, Sosnoff J. Commercially available accelerometry as an ecologically valid measure of ambulation in individuals with multiple sclerosis. Expert Rev Neurother. 2012;12(9):1079–1088.

21. Motl RW, Weikert M, Suh Y, et al. Accuracy of the actibelt(®) accelerometer for measuring walking speed in a controlled environment among persons with multiple sclerosis. Gait Posture. Elsevier B.V.; 2011 Sep 22;1–5.

22. Weikert M, Suh Y, Lane A, et al. Accelerometry is associated with walking mobility, not physical activity, in persons with multiple sclerosis. Med Eng Phys. Institute of Physics and Engineering in Medicine; 2011 Oct 1;1–8.

23. Sandroff BM, Klaren RE, Pilutti L a, Dlugonski D, Benedict RHB, Motl RW. Randomized controlled trial of physical activity, cognition, and walking in multiple sclerosis. J Neurol. 2014 Mar;261(2):363–372.

24. Moss-Morris R, McCrone P, Yardley L, van Kessel K, Wills G, Dennison L. A pilot randomised controlled trial of an Internet-based cognitive behavioural therapy self-management programme (MS Invigor8) for multiple sclerosis fatigue. Behav Res Ther. Elsevier Ltd; 2012 Jun;50(6):415–421.

25. Hobart J, Blight a. R, Goodman a., Lynn F, Putzki N. Timed 25-Foot Walk: Direct evidence that improving 20% or greater is clinically meaningful in MS. Neurology. 2013 Mar 27;

26. Pilutti L a, Dlugonski D, Sandroff BM, Klaren R, Motl RW. Randomized controlled trial of a behavioral intervention targeting symptoms and physical activity in multiple sclerosis. Mult Scler. 2014 Apr;20(5):594–601.

27. Motl RW, Pilutti LA, Learmonth YC, Goldman MD, Brown T. Clinical Importance of Steps Taken per Day among Persons with Multiple Sclerosis. 2013;8(9).
