# Supplementary material for: Feasibility of a smartphone app to enhance physical activity in progressive MS: a pilot randomized controlled pilot trial over three months
Source: PeerJ. 2020 Jun 23;8:e9303. doi: 10.7717/peerj.9303 (PMC7319035; doi:10.7717/peerj.9303)
Supplement: Supplemental Information 3 [file peerj-08-9303-s003.pdf]

CONSORT 2010 checklist of information to include when reporting a pilot or feasibility randomized trial in a journal or conference abstract

Item	Description	Reported on line number
Title	Identification of study as randomised pilot or feasibility trial	1
Authors *	Contact details for the corresponding author	3-28
Trial design	Description of pilot trial design (eg, parallel, cluster)	1
Methods		
Participants	Eligibility criteria for participants and the settings where the pilot trial was conducted	106-111
Interventions	Interventions intended for each group	103-106
Objective	Specific objectives of the pilot trial	97-99
Outcome	Prespecified assessment or measurement to address the pilot trial objectives**	133-136
Randomization	How participants were allocated to interventions	103
Blinding (masking)	Whether or not participants, care givers, and those assessing the outcomes were blinded to group assignment	113-114
Results		
Numbers randomized	Number of participants screened and randomised to each group for the pilot trial objectives**	114 / Figure 1
Recruitment	Trial status†	Not applicable
Numbers analysed	Number of participants analysed in each group for the pilot objectives**	114 / Figure 1
Outcome	Results for the pilot objectives, including any expressions of uncertainty**	204-212
Harms	Important adverse events or side effects	Not applicable
Conclusions	General interpretation of the results of pilot trial and their implications for the future definitive trial	291-295
Trial registration	Registration number for pilot trial and name of trial register	102
Funding	Source of funding for pilot trial	PeerJ section

**this item is specific to conference abstracts*

***Space permitting, list all pilot trial objectives and give the results for each. Otherwise, report those that are a priori agreed as the most important to the decision to proceed with the future definitive RCT.*

†For conference abstracts.
