# Supplementary material for: Feasibility of a smartphone app to enhance physical activity in progressive MS: a pilot randomized controlled pilot trial over three months
Source: PeerJ. 2020 Jun 23;8:e9303. doi: 10.7717/peerj.9303 (PMC7319035; doi:10.7717/peerj.9303)
Supplement: Supplemental Information 4 — Short information leaflet that served as the control condition in this pilot RCT. [file peerj-08-9303-s004.pdf]

Information sheet: Maintaining and improving health

Physical activity as an important building block for a healthy life

A healthy diet alone is not enough. Only those who exercise regularly can prevent lifestyle diseases. Without exercise, overweight, high blood pressure, diabetes and even concentration problems can occur. At the same time, sport promotes a sense of community and communicative skills as well as team spirit, ambition and self-esteem. Sports people are generally more balanced, healthier and live longer.

Lack of exercise as standard

Modern man often spends the whole day in the car, at the computer or in front of the television. But we are simply biologically wrongly equipped for this. In contrast to our ancestors, who walked for miles every day, our modern lifestyle tends to lack exercise and increase the risk of illness. Only 4 out of 10 healthy people move sufficiently according to international recommendations. In people with MS, it is only 2 out of 10.

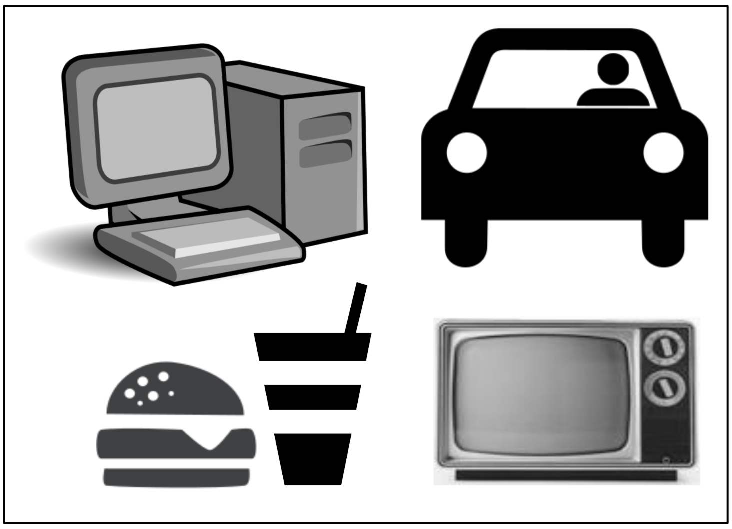

Lack of exercise has become standard

Not only overweight but also the risk of osteoporosis, diabetes, heart attack, stroke and dementia increases with lack of physical activity.

Exercise and chronic diseases

In addition, it is known that physical activity can have a beneficial effect on the body's self-healing powers in many chronic illnesses. For example, until a few years ago, patients with chronic heart failure were advised not to strain themselves. Today, however, regular, moderate exercise is recommended. This not only improves the resilience, but also noticeably improves the quality of life of those affected. Cancer patients also benefit from sport. The positive stress-reducing effect of sport on cancer has been shown several times. Patients suffered from severe exhaustion as a result of intensive cancer therapy. Regular training over several weeks not only improved the general condition, but also the psychological condition of those affected.

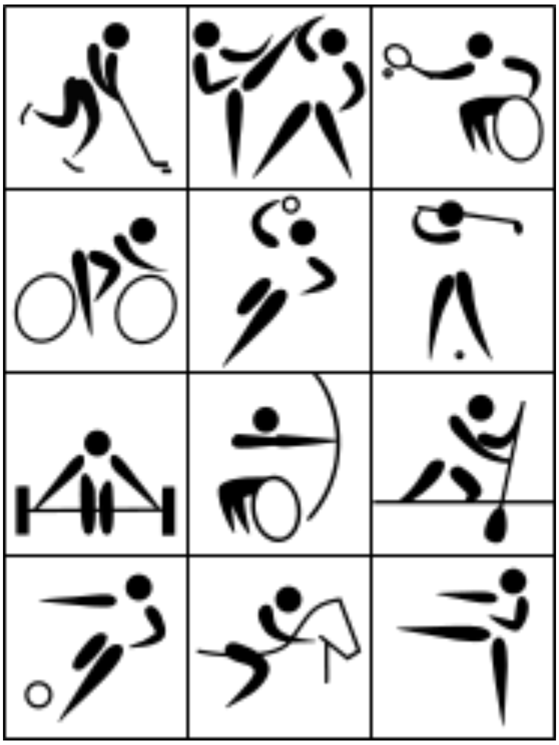

What sport doesn't matter.
The main thing is to move!

Be active!

Nowadays there is a clear connection between the increasing lack of exercise and the increasing number of mental disorders. Only those who exert themselves physically can relax, feel good afterwards and cope with stress better.

Those who exercise regularly stay physically and mentally fit. Whether swimming, walking, cycling or jogging is irrelevant - the main thing is to move!
